# Identification and molecular characterization of oat peptides implicated on coeliac immune response

**DOI:** 10.3402/fnr.v60.30324

**Published:** 2016-02-05

**Authors:** Isabel Comino, David Bernardo, Emmanuelle Bancel, María de Lourdes Moreno, Borja Sánchez, Francisco Barro, Tanja Šuligoj, Paul J. Ciclitira, Ángel Cebolla, Stella C. Knight, Gérard Branlard, Carolina Sousa

**Affiliations:** 1 Departamento de Microbiología y Parasitología, Facultad de Farmacia, Universidad de Sevilla, Sevilla, Spain; 2 Gastroenterology Unit, Antigen Presentation Research Group, Imperial College London & St Mark′s Hospital, Harrow, United Kingdom; 3 Hospital Universitario de La Princesa and Instituto de Investigación Sanitaria Princesa (IIS-IP), Centro de Investigación Biomédica en Red de Enfermedades Hepáticas y Digestivas (CIBEREHD), Madrid, Spain; 4 INRA UMR-1095, Clermont-Ferrand, France; 5 Nutrition and Bromatology Group, Department of Analytical and Food Chemistry, Food Science and Technology Faculty, University of Vigo-Ourense Campus, Ourense, Spain; 6 Instituto de Agricultura Sostenible (CSIC), Córdoba, Spain; 7 Division of Diabetes and Nutritional Sciences, King's College London, Gastroenterology, The Rayne Institute, St Thomas’ Hospital, London, United Kingdom; 8 Biomedal S.L., Sevilla, Spain

**Keywords:** coeliac disease, oats, immune response, gluten-free diet

## Abstract

**Background:**

Oats provide important nutritional and pharmacological properties, although their safety in coeliac patients remains controversial. Previous studies have confirmed that the reactivity of the anti-33-mer monoclonal antibody with different oat varieties is proportional to the immune responses in terms of T-cell proliferation. Although the impact of these varieties on the adaptive response has been studied, the role of the dendritic cells (DC) is still poorly understood. The aim of this study is to characterize different oat fractions and to study their effect on DC from coeliac patients.

**Methods and results:**

Protein fractions were isolated from oat grains and analyzed by SDS–PAGE. Several proteins were characterized in the prolamin fraction using immunological and proteomic tools, and by *Nano-LC-MS/MS*. These proteins, analogous to α- and γ-gliadin-like, showed reactive sequences to anti-33-mer antibody suggesting their immunogenic potential. That was further confirmed as some of the newly identified oat peptides had a differential stimulatory capacity on circulating DC from coeliac patients compared with healthy controls.

**Conclusions:**

This is the first time, to our knowledge, where newly identified oat peptides have been shown to elicit a differential stimulatory capacity on circulating DC obtained from coeliac patients, potentially identifying immunogenic properties of these oat peptides.

Coeliac disease (CD) is an autoimmune disorder developed in genetically (HLADQ2/8) predisposed individuals and caused by a permanent intolerance to gluten contained in some cereals, such as wheat, rye, and barley, that lead to a chronic inflammation of the small intestine (1–3).

The most accepted model for explaining CD immunopathogenesis is the two-signal model (4) characterized by a first innate immune response followed by a secondary antigen-specific adaptive response. Some peptides like the 19-mer gliadin peptide trigger an innate immune response (5) mainly characterized by the production of IL-15 by epithelial cells. The result is the disruption of the epithelial barrier, by increasing the permeability and inducing enterocyte apoptosis (6). As a consequence, immuno-adaptive peptides, like the 33-mer gliadin peptide, can now reach the lamina propria where they are deaminated by the enzyme tissue transglutaminase. Such deamidation provides a negative load to gliadin peptides and hence enhance their affinity to fit in the HLA-DQ2/8 bound, which is also the ‘susceptibility gene’ in CD, expressed on the surface of dendritic cells (DC) (7–9). DC are indeed the most potent antigen-presenting cells of the immune system as they promote differentiation of pro-inflammatory antigen-specific effector T-cell at the time that they also direct them to the target tissue via homing marker imprinting (10, 11). DC are therefore central in CD pathogenesis as they present a gluten antigen to T-cells (12) driving progression of the pro-inflammatory antigen-specific adaptive immune response which will turn into the symptomatology of the disease.

The gluten-free diet (GFD) is the basis of all the present treatments for CD, after which the immune response is abrogated leading to clinical remission of the disease (13, 14). Recently, the gluten-free products market has witnessed a revolution due to an increased incidence of CD coupled with the fact that it is not only coeliac and gluten-sensitive patients consuming these products but also individuals not affected by those pathologies (15). Therefore, oats are of particular interest to all GFD consumers because they could improve the nutritional value of the GFD given their rich nutritional value and a considerably high protein concentration. Oats also contain a number of important minerals, lipids, β-glucan, a mixed-linkage polysaccharide, which forms an important part of oat dietary fiber, and also contains various other phytoconstituents. Finally, oats also have different pharmacological activities including antioxidant, anti-inflammatory, antidiabetic, anticholesterolemic, and many others. Therefore, all these properties have led to wider appreciation of oats as valuable human food (16).

Studies on the suitability of oats for patients with CD showed contradictory results. While some studies suggested that oats are safe for CD patients (17–20), others have revealed that oats can trigger an immune reaction in these patients (18, 21–23), including activation of mucosal T-cells and subsequent gut inflammation ultimately leading to villous atrophy (22). Indeed, CD patients have circulating anti-avenin antibodies (24, 25), while a recent study revealed that dietary oats altered the mRNA immune status of the intestinal mucosa suggesting T-cell activation and leaky tight junctions (26). These contradictory results regarding oats safety for CD patients might be explained by the fact that the oat varieties used in the different studies were different. Oats include numerous varieties, differing in the prolamin genes and hence in the resulting amino acid sequences showing different immunoreactivities associated with toxic prolamins (27, 28). In previous studies, our group has reported the utility of the G12 monoclonal antibody (moAb) against the main immunogenic epitope of the α-2 gliadin, 33-mer peptide, for detecting oat varieties that are potentially toxic for CD patients. Indeed, the reactivity with the anti-33-mer moAb of the different oat varieties correlated with T-cell proliferation and interferon gamma production of blood T-cells isolated from such patients (29).

In the present study, we have characterized the different protein fractions of oat prolamins and have identified several reactive sequences to anti-33-mer moAb, analogous to α- and γ-gliadin-like, with immunogenic potential for CD patients. Moreover, some of the peptides from these subunits, obtained from *Nano-LC-MS/MS* data, induced specific activation of circulating DC obtained from CD patients on the GFD, as opposed to those from healthy controls (HC), confirming therefore their immunogenic potential.

## Materials and methods

### Oat sample preparation

Oats (*Avena sativa* L.) from cultivars designated OE717, OA729, OM719, OC723, OH727, and OL715 (obtained from Spanish and Australian commercial sources) were used in this work. These cultivars were chosen based on their previously reported CD toxicity (29).

### DNA extraction

DNA extraction from oat seeds was performed using a modified cetyltrimethylammonium bromide (CTAB) method. DNA concentrations were determined by UV absorption. The purity of the DNA solution was assessed by the 260/280 nm absorption ratio. Oligonucleotides from wheat, barley, rye, and oats were used. This protocol and oligonucleotides have been characterized in detail in previous studies from our laboratory (29).

### Protein extraction and quantification

Oat flours were prepared by grinding the dehusked kernels. Wholemeal flour (100 mg) was used for the sequential extraction of the albumins, globulins, avenins, and glutenins according to Osborne (30) and Marion et al. (31).

### Albumin extraction

The albumins were extracted with cold water with continuous mixing at 4°C for 90 min. The mixture was centrifuged (8,000*g*, 20 min) and the proteins in the supernatant were precipitated with two volumes of acetone at −20°C. The pellet was then washed three times with acetone and dried at room temperature.

### Globulin extraction

Globulins were extracted from the pellet with a salt solution (Tris-HCl 50 mM, NaCl 1 M, pH 8.5) in continuous mixing at 4°C for 1 h 30 min. Similarly, the mixture was centrifuged and the proteins in the supernatant were recovered with acetone.

### Avenins extraction

To extract avenins, three washes were performed with cold water on the pellet described above for 5 min with continuous vortexing. The samples were treated with 70% ethanol (v/v) for 60 min with continuous mixing, followed by centrifugation (10,000*g*, 5 min). This step was repeated three times to remove most of the avenins. The supernatants were pooled and incubated overnight at 65°C to recover the avenins.

### Glutenins extraction

The glutenins in the pellet were extracted and reduced with 50% propanol-1-ol solution containing 1% w/v dithiothreitol (DTT) for 30 min with continuous mixing at 65°C, followed by centrifugation (10,000*g*, 5 min) (32).

### SDS–PAGE

Proteins (albumins, globulins, avenins, and glutenins) were resuspended in SDS–polyacrylamide gel electrophoresis (PAGE) loading buffer (45 mM Tris-HCl, pH 6.8, 50 mM DTT, 1% SDS, 10% glycerol, 0.001% bromophenol blue). Protein concentration was determined by Bradford assay (33). Proteins were separated by SDS–PAGE using 12.5% polyacrylamide gel using a Hoefer tank (GE Healthcare, Little Chalfont, Buckinghamshire, UK). Gels were stained with Coomassie Brilliant Blue (CBB).

### Acid PAGE

In order to identify a large number of avenins according to their relative mobility, an acid PAGE (A-PAGE) was performed following the method of Branlard et al. (34).

### 2-DE

For further characterization of avenins, two biological extracts with two replicates per extract were used. Isoelectric focusing (IEF) was performed using the IPGPhor II apparatus (GE Healthcare) on 13 cm Immobiline dry strips of 3–10 linear pH gradients. Passive re-hydration was performed overnight in a solution containing 7 M urea, 2 M thiourea, 70 mM DTT, 1% IPG buffer (pH 3–10), 4% CHAPS, 0.34% anti-protease, and 100 µg (analytical gel) or 1 mg (preparative gel) of the protein extract. IEF was carried out by applying a cumulative voltage of 30 and 60 kVh for analytical and preparative gel, respectively.

Following IEF, proteins were reduced for 15 min in an equilibration buffer containing 0.05 M Tris-HCl (pH 8.8), 6 M urea, 30% glycerol, 2% SDS, and 1% DTT, followed by alkylation for 15 min in the same buffer containing 2.5% iodoacetamide instead of DTT. The second dimension was performed using SDS–PAGE gels (12% T, 2.1% C) sealed with 0.5% agarose in SDS buffer on Hoefer vertical system (GE Healthcare). The migration conditions were 10 mA/gel for first 30 min then 35 mA/gel until the exit of the dye front. Gels were stained with CBB.

### Immunoblotting

For immunoblot analysis, protein samples were separated on a 12.5% SDS–PAGE or a 2-DE gel and then transferred onto a nitrocellulose membrane using a Hoefer TE77 semidry transfer blotter. The blotted membrane was incubated for 60 min at room temperature in blocking buffer consisting of 10 mM Tris-HCl (pH 7.6), 150 mM NaCl, 0.01% Tween 20 and 5% skimmed milk and then exposed to G12 moAb.

Anti-mouse IgG alkaline phosphatase A3562 and kit SIGMA fast (ref F4523) were used for detection according to the manufacturer instructions.

Gel images of 300 dpi and 16-bit grayscale pixel depth were obtained with G-800 (GE Healthcare) scanner and were analyzed using SameSpots v3.2 (Nonlinear Dynamics, Newcastle, UK). SameSpots applies highly accurate pixel-level alignment so that 2-D gels with secondary stained images including antibodies can be directly compared.

### In-gel digestion

Protein spots were excised from gels and de-stained with a solution containing 25 mM NH_4_HCO_3_, 5% ACN for 30 min and 25 mM NH_4_HCO_3_ 50% ACN twice for 30 min. After dehydration in 100% ACN for 10 min, the spots were dried. Briefly, 100 ng of chymotrypsin (Sigma, St. Louis, MO, USA), solution in Tris-HCl 100 mM, pH 7.8, CaCl_2_ 10 mM was added to the spots and digestion was performed at 37°C for 4–5 h. After centrifugation, peptides were extracted by adding 8 µL of ACN.

### Nano-LC-MS/MS analysis and database searching

For Nano-LC-ESI-MS/MS analysis, peptides mixtures were analyzed by online nanoflow liquid chromatography using the Ultimate 3000 RSLC (Dionex, Voisins le Bretonneux, France) with nanocapillary columns of 15 cm length×75 µm ID, 3 µm, 100Å (Acclaim PepMap100 C18, Dionex). The solvent gradient increased linearly from 4 to 50% acetonitrile in 0.5% formic acid at a flow rate of 300 nL/min for 30 min. The elute was then electrosprayed in positive-ion mode at 2.7 kV in a LTQ-VELOS mass spectrometer (Thermo Fisher Scientific, Courtaboeuf, France) through a nanoelectrospray ion source which was operated in a CID top 10 mode (i.e. one full scan MS and the 10 major peaks in the full scan were selected for MS/MS). Full-enhanced-scan MS spectra were acquired with 1 microscan (*m*/*z* 300–1,800). Dynamic exclusion was used with one repeat count and 50 sec exclusion duration. For MS/MS, isolation width for ion precursor was fixed at 2 *m*/*z*, and single-charged species were rejected; fragmentation used 37% normalized collision energy as the default activation of 0.25.

### Protein and peptide sequence analysis

Raw data files were processed using version Peaks 5.3 software with the EBI database (Taxonomy Viridiplantae, 1,023,819 entries). The following parameters were considered for the searches: Parent Mass Error Tolerance of 1.5 Da, Fragment Mass Error Tolerance of 0.8 Da, a maximum of one missed cleavage, and partial methionine oxidation and partial carbamidomethylation of cysteine. If the peaks score was statistically significant (*p*<0.05), the protein was considered valid. When proteins were identified from only two peptides, spectra were checked to assess their validity.

### Synthesis of peptides

Different peptides derived from avenin sequences obtained from the MS/MS data (Table 1) were supplied by Biomedal S.L. (Seville, Spain).

**Table 1:** Peptides derived from avenin sequences

Peptide	Sequence
*Gliadin-like avenins*	
QL6	QPQLQL
QQ6	QPQLQQ
PV10	PYPEQQEPFV
EF27	EQYQPYPEQQEPFVQQQPPFVQQEQPF
*Glutenin-like avenins*	
QL14	QQPFMQQQPFMQPL
QM27	QYQPYPEQQPFMQQQQPFMQPLLQQQM

### DC from peripheral blood

Human peripheral blood was collected from three HC with no known autoimmune or inflammatory diseases, allergies, or malignancies and three patients with CD following informed consent. All CD patients had been on a GFD for at least 6 months and had no clinical symptoms or positive serology at the time of sample taking. The study was approved by the ethic committee of St Thomas’ Hospital, London (United Kingdom) and written informed consent was obtained.

Peripheral blood mononuclear cells (PBMC) were obtained by centrifugation over Ficoll-Paque Plus (Amersham Biosciences, Chalfont St. Giles, UK). Human blood–enriched DC were subsequently enriched following NycoPrep™ centrifugation of overnight cultured PBMC in complete medium Dutch modified RPMI 1640 (Sigma-Aldrich, Dorset, UK) containing 100 U/mL penicillin/streptomycin, 2 mM L-glutamine, 50 µg/mL gentamicin (Sigma-Aldrich), and 10% fetal calf serum (TCS Cellworks, Buckingham, UK). This protocol has been characterized in detail in previous studies as a way to obtain fresh human blood–enriched DC (35–38). Blood-enriched DC were further cultured for 24 h in complete medium in the presence of oat peptides, 33-mer peptide, STp (peptide secreted by *Lactobacillus plantarum*) or LPS (lipopolysaccharide from *Escherichia coli*) (Sigma-Aldrich, St. Louis, MO, USA). The same molar concentration was used for all peptides to avoid the problem of epitope load between large and small peptides. Results were referred with a paired culture in basal medium which acted as an internal control.

### Proliferation assay

Freshly obtained PBMC from HC were suspended in MiniMACs buffer (PBS containing 0.5% BSA and 2 mM EDTA). T-cells were enriched by depletion of CD14, CD19, and HLA-DR-positive cells with immunomagnetic beads (Miltenyi Biotech, Bisley, UK) following manufacturer's instructions. An average of 94.91%±1.06 (mean±SD) T-cells was obtained following enrichment. T-cells were labeled with 10 µM 5-carboxyfluorescein diacetate succinimidyl ester (CFSE, Invitrogen Ltd, Paisley, UK) following manufacturer's instructions. CFSE-labeled T-cells (4×10^5^/well) were incubated for 5 days in U-bottomed 96-well microtiter plates with allogeneic blood DC at 0, 1, or 3%. Cells were washed twice in FACS buffer, fixed with 1% paraformaldehyde in 0.85% saline, and stored at 4°C prior to acquisition.

### Flow cytometry and data analysis

Cells were acquired on a FACSCantoII (BD Biosciences) flow cytometer (within 48 h). Data were analyzed using WinList 5.0 software (Verity, Topsham, Maine, USA). The percentage of proliferating T-cells was assessed via CFSE dilution of viable cells based on their forward and size properties as previously described (11).

### Statistical analysis

Results were analyzed in the GraphPad Prism statistical PC program (GraphPad Prism 6.0 Software, San Diego, CA) using two-way and one-way repeated measures ANOVA, and two-tailed paired tests. *P*-value<0.05 was considered significant.

## Results and discussion

### Protein characterization in different oat varieties

**Fig. 1:**
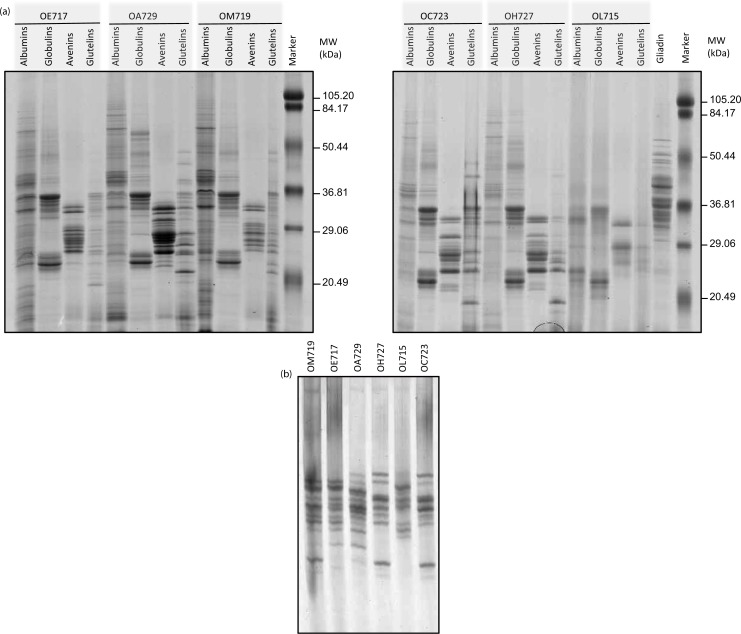
Protein pattern from six oat accessions. (a) Oat proteins (albumins, globulins, avenins, and glutelins) were analyzed by SDS–PAGE using 12.5% polyacrylamide gel and stained with Coomassie Brilliant Blue (CBB). (b) Avenins were analyzed by A-PAGE gel and stained with CBB. Oat accessions: OE717, OA729, OM719, OC723, OH727, and OL715. Lane MW, molecular weight markers (in kDa).

Protein fractions from six oat varieties previously described as potentially reactive for CD patients (29) were studied by SDS–PAGE. The purity of the oat seeds was tested by a visual examination, and PCR experiments discarded the presence of wheat, rye, and barley DNA in the samples. The protein patterns of these cereals were compared with those of gliadins (from wheat) and with a pre-stained molecular weight marker (Fig. 1a).

Proteins with a wide range of molecular weights ranging from <20 to 80 kDa were found in all albumin fractions of the different cultivars. Oat albumins, which are considered to be primarily enzymes, are a minor component with values ranging from 1 to 12% of total protein (39). Nevertheless, most of the protein fraction from the oat grains was soluble in buffered salt solutions and thus classified as globulins (40). These proteins (Fig. 1a) were mainly of two families, the first ranging from 20 to 37 kDa, and the second grouping around 50 kDa. Globulins appeared with the same distribution in all cultivars studied. Opposed to wheat gliadins, which have a typical size range of 29–70 kDa, oat avenins were smaller in size ranging from 20 to 36 kDa with weaker bands around 50–70 kDa. Another important feature of these proteins is that their protein patterns were diverse, confirming that polymorphism of avenin patterns was more heterogeneous than in the globulin fraction.

Concerning the glutenin fraction, a wide range in the molecular weight, ranging from 50 to even lower than 20 kDa, was also observed for these proteins. For all other accessions, the glutenin protein patterns showed a diversity and heterogeneity in both size and intensity of the subunits. Our findings confirmed therefore that oat grains contain a significant protein fraction that is insoluble in alcohol and soluble in denaturing/reducing solution composed mainly of low molecular weight glutenin subunits (LMW-GS)-like proteins (41).

Because of the diversity found in the alcohol-soluble fractions, in order to identify the relative mobility of these proteins, an acid PAGE (A-PAGE) was performed. The diversity of cereal prolamins is usually better resolved by using electrophoresis in aluminum lactate buffer (such as in A-PAGE). Moreover, A-PAGE allows oat prolamins to be separated according to the ratio ionic charge/molecular mass resulting in a better resolution as compared to the classic SDS–PAGE procedure. A large number of avenin subunits were observed (Fig. 1b). Differences in the band patterns were found for the studied varieties, showing a great diversity in the composition of avenins among the different cultivars. These results therefore confirm the presence of different avenins in the cultivars as they differ in both their size and ionic charge in acid pH. However, Spanish varieties OH727 and OC723 shared the same pattern of bands by A-PAGE. Likewise, two Australian accessions, OE717 and OM719, kept the same prolamin pattern but differed in two protein bands obtained in the lower region of the gel. These accessions probably have the same progenitors or related progenitors, or they may have independently evolved from accessions with small differences between them.

### Evaluating immunotoxic proteins in the alcohol-soluble fraction from oat seeds

**Fig. 2:**
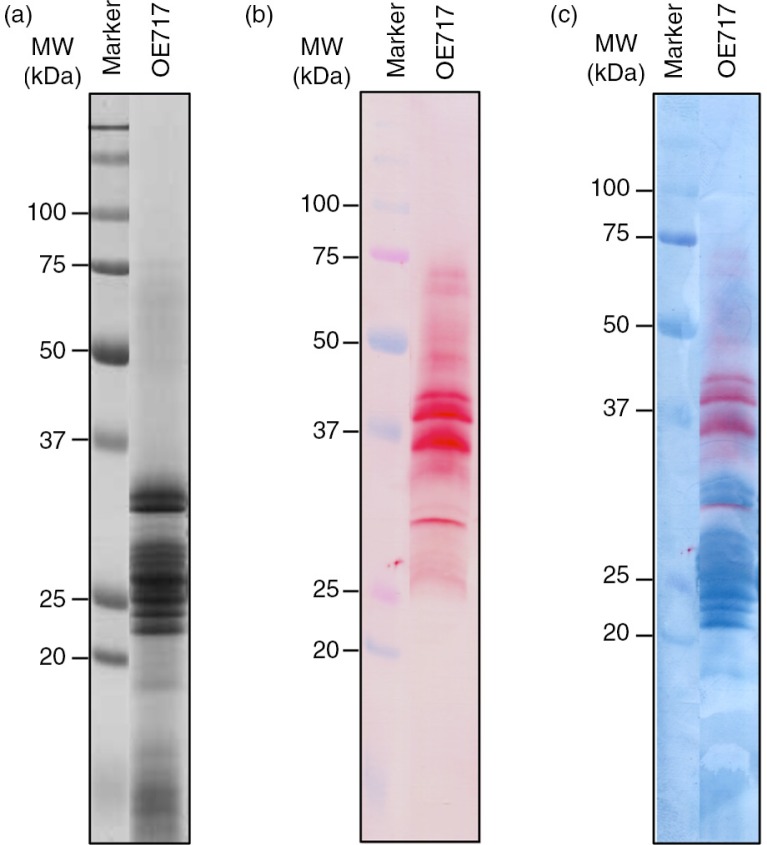
Electrophoresis and immunoblot analysis of avenin protein extracts from oat accession OE717. (a) Proteins were separated by SDS–PAGE using 12.5% polyacrylamide gel and stained with Coomassie Brilliant Blue (CBB), (b) then transferred onto a nitrocellulose membrane and then exposed to G12 moAb. (c) Dual double membrane staining, first using G12 moAb and then nigrosin, distinguished the total avenin fraction versus G12-specific proteins. Lane MW, molecular weight markers (in kDa).

In order to get further insights into the proteomic and immunological properties of the avenin proteins previously described, and their implications in CD pathogenesis, we focused on accession OE717 as it was described with high immunogenic effect of CD patients (29). The avenin extract from this accession was studied by western blot using staining techniques which reveal in the membrane at the same time both the antibody-recognized proteins and the total proteins as separated by SDS–PAGE. A dual staining, first with the moAb and then nigrosin, distinguished the total avenin fraction versus specific proteins (Fig. 2). Reactive proteins appeared in the region of 25–37 kDa (major bands according to results obtained by SDS–PAGE); however, anti-33-mer moAb also recognized other minority oat prolamins with higher molecular weights. The latter may be avenin dimers and/or oat prolamins not yet labeled with higher molecular weights.

**Fig. 3:**
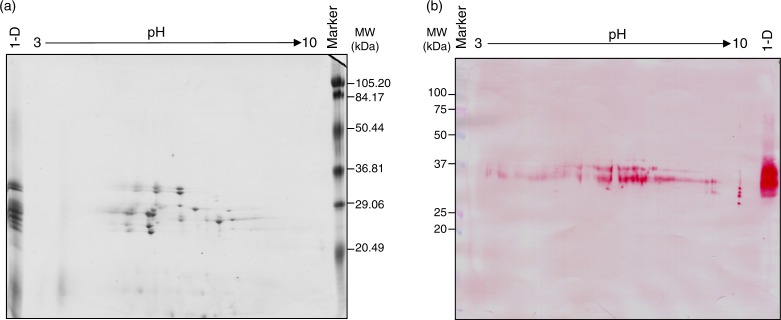
Bi-dimensional analysis of avenin protein extracts from oat accession OE717. (a) Proteins were separated by isoelectric focusing (IEF) and SDS–PAGE, and then stained using Coomassie Brilliant Blue (CBB). (b) 2-D gel electrophoresis was transferred onto a nitrocellulose membrane and exposed to G12 moAb. Lane 1-D: avenin protein extract separated by SDS–PAGE. Lane MW, molecular weight markers (in kDa).

When avenins from OE717 variety were separated in more detail by 2-DE, a single band on 1-DE typically yielded more than one protein spot (Fig. 3a). Avenin fraction was separated by 2-DE and made visible by CBB staining. The 2-DE gels of these proteins revealed spots with relative molecular masses ranging from 35 to 20 kDa and a p*I* between 3 and 10. Figure 3b showed proteome map obtained after immunoblotting by anti-33mer moAb. Immunoreactive spots were observed ranging from 40 to 25 kDa on the western blot.

Among all the identified bands and spots, we next focused on the most reactive one as revealed by their immunoblotting intensity (Fig. 2b and 3b). Following overlaying of the nigrosin-stained membrane, the immunoblot and the corresponding CBB stained gel, bands (1-DE) and spots (2-DE) (as revealed by G12 moAb from the CBB stained gel) were excised and mass spectrometry analysis subsequently applied. A total of 16 reactive proteins were identified, all belonging to the family of prolamins, specifically avenin proteins (Table 2). We further confirmed by BLAST searches, using *Multiple Protein Alignment Tool* algorithms, that all the proteins identified belong to fractions previously termed α- and γ-gliadin-like. However, some bands and spots could not be identified due to lack of information from oat proteins in available databases.

**Table 2:** List of identified proteins from 1-D and 2-D gels of avenin protein extract from oat accession OE717

Sample^a^	Accession	Cov (%)	Peptides	Mass	Description
A6	TR:G8ZCU1_9POAL	60	4	27344.3008	G8ZCU1 Avenin protein (Fragment) OS=*Avena prostrata* GN=avenin PE=4 SV=1
	TR:G8ZCT4_9POAL	59	4	29672.8789	G8ZCT4 Avenin protein (Fragment) OS=*Avena longiglumis* GN=avenin PE=4 SV=1
	TR:F2Q9W5_AVESA	53	3	31613.2422	F2Q9W5 Avenin protein (Fragment) OS=*Avena sativa* GN=avenin PE=4 SV=1
	SP:AVEF_AVESA	100	2	5213.6143	Q09097 Avenin-F (Fragment) OS=*Avena sativa* PE=1 SV=1
A7	TR:F2Q9W5_AVESA	55	6	31613.2422	F2Q9W5 Avenin protein (Fragment) OS=*Avena sativa* GN=avenin PE=4 SV=1
	TR:Q09072_AVESA	61	6	25471.4941	Q09072 Avenin (Precursor) OS=*Avena sativa* PE=2 SV=1
	TR:G8ZCU1_9POAL	57	6	27344.3008	G8ZCU Avenin protein (Fragment) OS=*Avena prostrata* GN=avenin PE=4 SV=1
	TR:G8ZCT1_9POAL	51	4	28834.0137	G8ZCT avenin protein (Fragment) OE=*Avena strigosa* GN=avenin PE=4 SV=1
	SP:AVEE_AVESA	66	3	21036.1562	Q09114 Avenin-E OS=*Avena sativa* PE=1 SV=1
	TR:G8ZCT5_9POAL	53	4	28018.0723	G8ZCT5 Avenin protein (Fragment) OS=*Avena damascena* GN=avenin PE=4 SV=1
	TR:G8ZCT6_9POAL	53	4	28018.0723	G8ZCT6 Avenin protein (Fragment) OS=*Avena strigosa* GN=avenin PE=4 SV=1
	TR:G8ZCT7_9POAL	53	4	28018.0723	G8ZCT7 Avenin protein (Fragment) OS=*Avena canariensis* GN=avenin PE=4 SV=1
	TR:G8ZCV9_9POAL	57	4	26660.6230	G8ZCV9 Avenin protein (Fragment) OS=*Avena murphyi* GN=avenin PE=4 SV=1
	TR:G8ZCW4_9POAL	57	3	24318.9219	G8ZCW4 Avenin protein (Fragment) OS=*Avena macrostachya* GN=avenin PE=4 SV=1
	TR:F2Q9W3_AVESA	52	2	25732.9043	F2Q9W3 Avenin protein (Fragment) OS=*Avena sativa* GN=avenin PE=4 SV=1
	TR:G8ZCW2_9POAL	16	2	23778.5078	G8ZCW2 Avenin protein (Fragment) OS=*Avena ventricosa* GN=avenin PE=4 SV=1
	TR:G8ZCW5_9POAL	16	2	23778.5078	G8ZCW5 Avenin protein (Fragment) OS=*Avena murphyi* GN=avenin PE=4 SV=1
	TR:G8ZCV7_9POAL	16	2	24500.3652	G8ZCV7 Avenin protein (Fragment) OS=*Avena magna* GN=avenin PE=4 SV=1
	TR:G8ZCV8_9POAL	16	2	24500.3652	G8ZCV8 Avenin protein (Fragment) OS=*Avena mnurphyi* GN=avenin PE=4 SV=1
A8	TR:G8ZCW3_9POAL	52	6	27387.3730	G8ZCW3 Avenin protein (Fragment) OS=*Avena insularis* GN=avenin PE=4 SV=1
	TR:G8ZCT8_9POAL	50	6	28205.2715	G8ZCT8 Avenin protein (Fragment) OS=*Avena canariensis* GN=avenin PE=4 SV=1
	TR:G8ZCT9_9POAL	50	6	28205.2715	G8ZCT9 Avenin protein (Fragment) OS=*Avena strigosa* GN=avenin PE=4 SV=1
	TR:F2Q9W5_AVESA	45	6	31613.2422	F2Q9W5 Avenin protein (Fragment) OS=*Avena sativa* GN=avenin PE=4 SV=1
	TR:G8ZCU1_9POAL	45	6	27344.3008	G8ZCU1 Avenin protein (Fragment) OS=*Avena prostrata* GN=avenin PE=4 SV=1
	TR:G8ZCU6_9POAL	45	4	24307.0723	G8ZCU6 Avenin protein (Fragment) OS=*Avena prostrata* GN=avenin PE=4 SV=1
	TR:G8ZCT2_9POAL	44	4	24435.2031	G8ZCT2 Avenin protein (Fragment) OS=*Avena prostrata* GN=avenin PE=4 SV=1
	TR:F2Q9W4_AVESA	48	4	24631.5215	F2Q9W4 Avenin protein (Fragment) OS=*Avena **s**ativa* GN=avenin PE=4 SV=1
	SP:AVEE_AVESA	52	4	21036.1562	Q09114 Avenin-E OS=*Avena **s**ativa* PE=1 SV=1
	TR:Q09072_AVESA	53	4	25471.4941	Q09072 Avenin (Precursor) OS=*Avena satival* PE=2 SV=1
	TR:G8ZCW4_9POAL	42	4	24318.9219	G8ZCW4 Avenin protein (Fragment) OS=*Avena macrostachya* GN=avenin PE=4 SV=1
	TR:F4MJY1_AVESA	40	4	25600.3555	F4MJY1 Avenin protein (Fragment) OS=*Avena sativa* GN=avenin PE=2 SV=1
	TR:G8ZCW0_9POAL	37	4	26011.8594	G8ZCW0 Avenin protein (Fragment) OS=*Avena murphyi* GN=avenin PE=4 SV=1
	TR:G8ZCU2_9POAL	55	4	24137.8320	G8ZCU2 Avenin protein (Fragment) OS=*Avena longiglumis* GN=avenin PE=4 SV=1
	TR:G8ZCU3_9POAL	55	4	24137.8320	G8ZCU3 Avenin protein (Fragment) OS=*Avena strigosa* GN=avenin PE=4 SV=1
	TR:G8ZCU4_9POAL	55	4	24111.7949	G8ZCU4 Avenin protein (Fragment) OS=*Avena strigosa* GN=avenin PE=4 SV=1
	TR:G8ZCU5_9POAL	44	4	23093.7402	G8ZCU5 Avenin protein (Fragment) OS=*Avena longiglumis* GN=avenin PE=4 SV=1
A9	SP:AVE3_AVESA	60	7	25275.4844	P80356 Avenin-3 OS=*Avena sativa* PE=1 SV=1
	TR:Q2EPY2_AVESA	65	7	24258.3320	Q2EPY2 Avenin OS=*Avena sativa* PE=4 SV=1
	TR:G8ZCU1_9POAL	51	6	27344.3008	G8ZCU1 Avenin protein (Fragment) OS=*Avena prostrata* GN=avenin PE=4 SV=1
	TR:G8ZCW3_9POAL	54	6	27387.3730	G8ZCW3 Avenin protein (Fragment) OS=*Avena insularis* GN=avenin PE=4 SV=1
	SP:AVEE_AVESA	66	5	21036.1562	Q09114 Avenin-E OS=*Avena sativa* PE=1 SV=1
	TR:Q09072_AVESA	51	4	25471.4941	Q09072 Avenin (Precursor) OS=*Avena sativa* PE=2 SV=1
	TR:G8ZCV7_9POAL	62	4	24500.3652	G8ZCV7 Avenin protein (Fragment) OS=*Avena magna* GN=avenin PE=4 SV=1
	TR:G8ZCV8_9POAL	53	4	24500.3652	G8ZCV8 Avenin protein (Fragment) OS=*Avena murphyi* GN=avenin PE=4 SV=1
	TR:G8ZCU0_9POAL	54	4	23039.7891	G8ZCU0 Avenin pratein (Fragment) OS=*Avena damascena* GN=avenin PE=4 SV=1
	TR:G8ZCW4_9POAL	51	5	24318.9219	G8ZCW4 Avenin protein (Fragment) OS=*Avena macrostachya* GN=avenin PE=4 SV=1
	TR:G8ZCW0_9POAL	45	5	26011.8594	G8ZCW0 Avenin protein (Fragment) OS=*Avena murphyi* GN=avenin PE=4 SV=1
AS1	TR:G8ZCU1_9POAL	57	10	27344.3008	G8ZCU1 Avenin protein (Fragment) OS=*Avena prostrataa* GN=avenin PE=4 SV=1
	TR:F2Q9W5_AVESA	50	9	31613.2422	F2Q9W5 Avenin protein (Fragment) OS=*Avena **s**ativa* GN=avenin PE=4B SV=1
	TR:G8ZCT1_9POAL	53	6	28834.0137	G8ZCT1 Avenin protein (Fragment) OS=*Avena strigosa* GN=avenin PE=4 SV=1
	SP:AVEF_AVESA	84	3	5213.6143	Q09097 Avenin-F (Fragment) OS=*Avena sativa* PE=1 SV=1
AS3	TR:F2Q9W5_AVESA	48	6	31613.2422	F2Q9W5 Avenin protein (Fragment) OS=*Avena sativa* GN=avenin PE=4 SV=1
	TR:G8ZCU1_9POAL	50	6	27344.3008	G8ZCU1 Avenin protein (Fragment) OS=*Avena prostrata* GN=avenin PE=4 SV=1
	TR:G8ZCW4_9POAL	51	6	24318.9219	G8ZCW4 Avenin protein (Fragment) OS=*Avena macrostachya* GN=avenin PE=4 SV=1
	TR:F4MJY1_AVESA	57	6	25600.3555	F4MJY1 Aveninl protein (Fragment) OS=*Avena sativa* GN=avenin PE=2 SV=1
	TR:G8ZCW0_9POAL	56	6	26011.8594	G8ZCW0 Avenin protein (Fragment) OS=*Avena murphyi* GN=avenin PE=4 SV=1
	TR:Q09072_AVESA	55	4	25471.4941	Q09072 Avenin (Precursor) OS=*Avena sativa* PE=2 SV=1
	SP:AVEE_AVESA	55	5	21036.1562	Q09114 Avenin-E OS=*Avena sativa* PE=1 SV=1
	SP:AVEF_AVESA	67	3	5213.6143	Q09097 Avenin-F (Fragment) OS=*Avena sativa* PE=1 SV=1

a Samples obtained from the 1-D and 2-D gel spots.

### Circulating DC from CD patients react to avenin peptides

CD pathogenesis is driven both by the innate and the adaptive immune system (1, 4, 42). Although the impact of gluten peptides on the adaptive immune system has been studied in much detail, the role of the innate immune response cells, including monocytes/macrophages and DC, is still poorly understood.

DC are key actors in the connection between innate immunity and adaptive immunity responses. Furthermore, they are described as ‘decision makers’ to commit tolerance or immunity (12, 43), yet information on DC in CD pathogenesis is scarce (8, 44–47). Moreover, most of the studies which have investigated the effect of gliadin and/or its derived peptides on DC phenotype and/or function have usually focused on monocyte-derived DC, generated following 5–7 *in vitro* culture of monocytes in the presence of IL-4 and GM-CSF (48–53), which although essential to further our understanding of human DC do not always resemble the properties of circulating DC (54, 55). Moreover, these studies usually focus on the effect of gliadin and/or its derived peptides on monocyte-derived DC from HC, usually avoiding a comparison with those obtained from CD patients. Contrary to those studies, here we decided to study the effect of these newly identified no-wheat oat peptides on circulating DC obtained from both HC and CD patients.

To determine the stimulatory capacity of the novel oat peptides, DC were pulsed with peptides QL6, QQ6, PV10, EF27, QL14, and QM27 of different sizes that were found having a homology to gliadin-like avenins (Table 1) and glutenin-like avenins. These peptides had proline-rich sequences and glutamine residues resembling wheat gluten sequences. Peptides PV10 and EF27 also carried a T-cell epitope recognized by CD4^+^ T-cells previously described by Sollid et al. (56): DQ2.5-ave-1a epitope with glutamic acid to glutamine conversion at position 6. As positive control for stimulation, DC were pulsed with 33-mer peptide, the immunodominant antigen for CD (57) or with LPS. In addition to the basal (unstimulated) internal negative controls, DC were also pulsed with microbiota-derived STp, previously described to induce regulatory effects on DC in HC without affecting their stimulatory capacity (38).

**Fig. 4:**
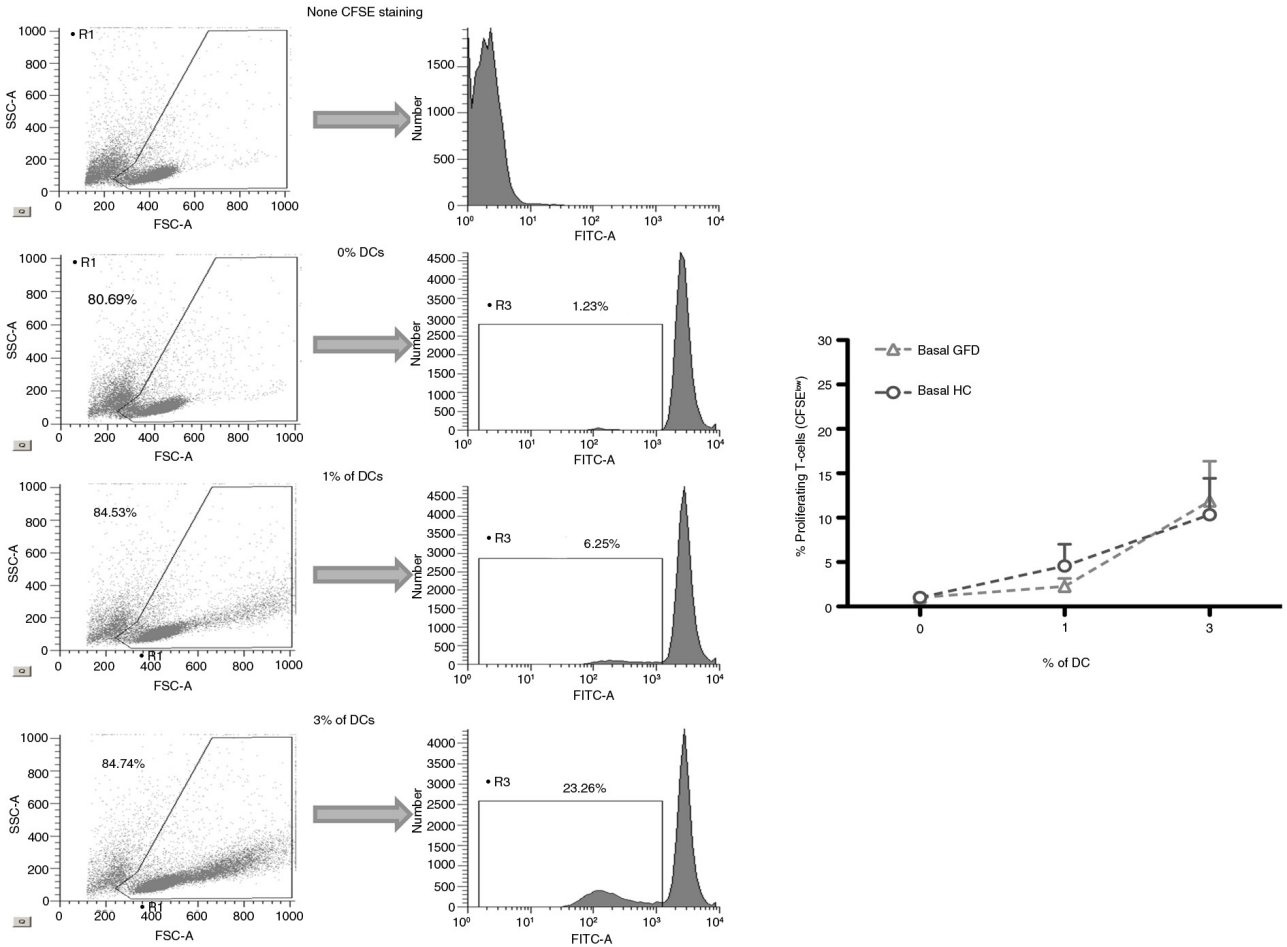
Identification of dividing T-cells after dendritic cell stimulations. Blood CFSE-labeled T-cells (400,000) from healthy controls (HC) were cultured for 5 days with different doses (0, 1, and 3%) of allogeneic blood–enriched DC obtained from HC and gluten-free diet (GFD) coeliac disease patients. Live T-cells were subsequently identified by flow cytometry based on the forward (FSC-A) and side (SSC-A) scatter properties. DC stimulatory capacity was assessed based on T-cell proliferation determined via CFSE dilution (determined on the FITC channel) compared with unstimulated T-cells (cultured in the absence of DC). A second negative control included the culture of CFSE-negative T-cells. Histograms are representative of several independent experiments performed with similar results. Pooled results are displayed on the right plot.

In order to exclude any potential effect of the ongoing inflammation on the profile of circulating DC, and therefore on their peptide response following *in vitro* challenge, experiments were performed with blood-enriched DC from HC and GFD-CD patients with no clinical symptoms and negative serology at the time of blood extraction. Following antigen-pulsing and subsequent co-culture with T-cells, DC from both HC and GFD-CD patients induced dose-dependent proliferative responses of CFSE-labeled allogeneic T-cells (determined as CFSE dilution by responding or dividing T-cells, Fig. 4) with no differential effect produced by blood DC from the groups irrespective of any differential basal stimulatory status between the groups.

**Fig. 5:**
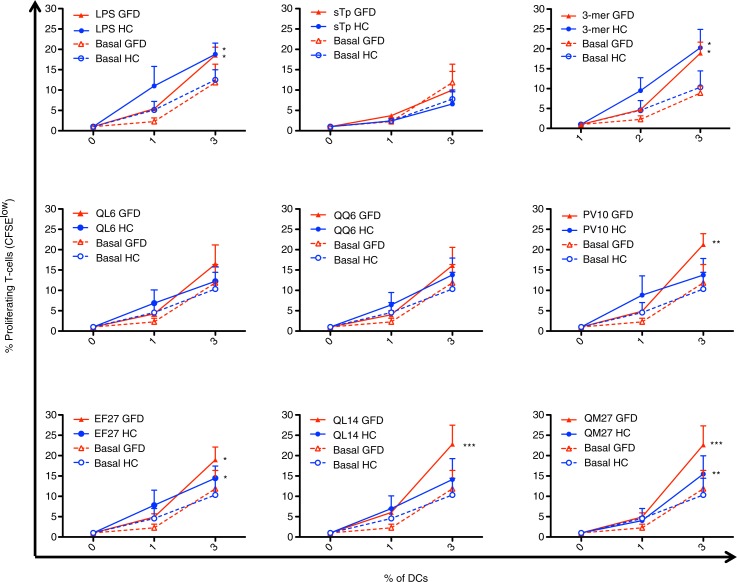
Effect of oat peptides on the stimulatory capacity of DCs for allogeneic T-cells in CD. DC stimulatory capacity for CFSE-labeled allogeneic T-cells was determined as in Fig. 4. DC stimulatory capacity from both gluten-free diet coeliac patients (GFD, red lines) and healthy controls (HC, blue lines) was determined following 24-h pulsing with different stimuli as detailed in the graphs. Two-way ANOVA repeated was applied on pulsed DC (GFD or HC) compared with their basal paired counterparts. *P*-values <0.05 were considered as statistically significant (*p<0.05; **p<0.01; ***p<0.001).

The effect of the previously newly identified non-wheat oat peptides on DC from both HC and GFD-CD patients was studied next (Fig. 5). LPS increased stimulatory capacity of blood DC derived from both HC and GFD-CD while STp did not induce any change in DC stimulatory status as previously described (Fig. 5) (38). When pulsed with the immunodominant 33-mer peptide, DC from both HC and GFD-CD patients increased their stimulatory capacity for T-cells in agreement with previous observations of an ‘ex-vivo’ gluten-challenge biopsy-culture model (58). Having confirmed that *in vitro* pulsing modulates DC stimulatory capacity from both HC and GFD-CD patients, we studied whether the newly identified oat peptides displayed any differential effect on DC from the groups. Our findings revealed that oat peptides could be divided into three groups based on 1) their lack of stimulatory effect on DC (peptides QL6 and QQ6); 2) increase of DC stimulatory capacity from both HC and GFD-CD patients (peptides EF27 and QM27); and 3) peptides which specifically upregulated DC stimulatory capacity from GFD-CD patients but not from HC (peptides PV10 and QL14). A closer look into these oat peptides’ properties revealed that peptides QL6 and QQ6 (which did not have any stimulatory effect) were the smallest of the studied peptides (each with six residues). On the contrary, peptides EF27 and QM27 (which induced proliferative responses of T-cells stimulated by both HC and GFD-CD) were larger peptides (each with 27 residues); these peptides were of a size similar to the 33-mer peptide which had also activated DC from both HC and GFD-CD patients. This is in agreement with similar observations where large gliadin-derived peptides induced DC activation from non-CD patients (49, 50, 52). Finally, peptides PV10 and QL14 were unique in their capacity to specifically activate DC from GFD-CD patients but not those from HC. In contrast to the two other groups, peptides PV10 and QL14 had intermediate sizes (PV10: 10 residues; QL14: 14 residues). These differences between the differential stimulatory capacity of the peptides on DC from HC and GFD-CD are not likely to be due to different epitope loads derived from their differential size as DC pulsing was performed at the same molar concentration. It seems, therefore, that DC capacity to trigger T-cell proliferative responses is not only dependent on the source of the peptides but also on their size and their possible differential intracellular processing. Thus, small peptides like QL6 and QQ6 (six residues each) would fail to activate DC while large gluten peptides (peptides EF27 and QM27 – of 27 and 33 residues, respectively) would induce DC maturation in both HC and GFD-CD. Nevertheless, gluten peptides with the appropriate size and disposition of amino acids–peptides PV10 (10 residues) and/or QL14 (14 residues) are likely to go through a differential endocytic pathway which may end in a different peptide processing capacity elicited by DC from HC and CD patients. Future studies will identify the specific mechanisms which make circulating DC form CD patients unique on their capacity to process and present these intermediate peptides to the T-cells, which may therefore provide valuable information for development of novel biomarkers for CD diagnosis and/or monitoring. Indeed, given that intestinal DC are likely to be derived from their circulating precursors (59) it is quite likely that intestinal DC will also display such differential processing capacity. If so, a deeper characterization of the differential peptide processing capacity elicited by intestinal DC from CD patients and non-CD controls may provide novel information regarding CD pathogenesis which may lead to development of novel therapies for such patients.

## Conclusions

Our findings exhibited the structural complexity and large differences among oat proteins. More specifically, we showed that oats is composed of a large number of avenin subunits. These proteins belong to fractions termed α- and γ-gliadin-like and some of which were reactive for anti-33-mer moAb. Moreover, our study has shown the existence of new potentially toxic peptides for coeliac patients. These peptides were able to activate circulating DC from coeliac patients, identifying, therefore, their immunogenic properties.
